# Validation of the physiological background correction method for the suppression of the spill-in effect near highly radioactive regions in positron emission tomography

**DOI:** 10.1186/s40658-018-0233-8

**Published:** 2018-12-05

**Authors:** Mercy I. Akerele, Palak Wadhwa, Jesus Silva-Rodriguez, William Hallett, Charalampos Tsoumpas

**Affiliations:** 1 Biomedical Imaging Science Department, School of Medicine, University of Leeds, Leeds, West Yorkshire UK; 2 Invicro, Hammersmith Hospital, London, UK; 3 Molecular Imaging Research Group, Health Research Institute of Santiago de Compostela (IDIS), Santiago de Compostela, Galicia, Spain

**Keywords:** PET, Post-filtering, Reconstruction, Background correction, Lesion contrast

## Abstract

**Background:**

Positron emission tomography (PET) imaging has a wide applicability in oncology, cardiology and neurology. However, a major drawback when imaging very active regions such as the bladder is the spill-in effect, leading to inaccurate quantification and obscured visualisation of nearby lesions. Therefore, this study aims at investigating and correcting for the spill-in effect from high-activity regions to the surroundings as a function of activity in the hot region, lesion size and location, system resolution and application of post-filtering using a recently proposed background correction technique. This study involves analytical simulations for the digital XCAT2 phantom and validation acquiring NEMA phantom and patient data with the GE Signa PET/MR scanner. Reconstructions were done using the ordered subset expectation maximisation (OSEM) algorithm. Dedicated point-spread function (OSEM+PSF) and a recently proposed background correction (OSEM+PSF+BC) were incorporated into the reconstruction for spill-in correction. The standardised uptake values (SUV) were compared for all reconstruction algorithms.

**Results:**

The simulation study revealed that lesions within 15–20 mm from the hot region were predominantly affected by the spill-in effect, leading to an increased bias and impaired lesion visualisation within the region. For OSEM, lesion SUV_max_ converged to the true value at low bladder activity, but as activity increased, there was an overestimation as much as 19% for proximal lesions (distance around 15–20 mm from the bladder edge) and 2–4% for distant lesions (distance larger than 20 mm from the bladder edge). As bladder SUV increases, the % SUV change for proximal lesions is about 31% and 6% for SUV_max_ and SUV_mean_, respectively, showing that the spill-in effect is more evident for the SUV_max_ than the SUV_mean_. Also, the application of post-filtering resulted in up to 65% increment in the spill-in effect around the bladder edges. For proximal lesions, PSF has no major improvement over OSEM because of the spill-in effect, coupled with the blurring effect by post-filtering. Within two voxels around the bladder, the spill-in effect in OSEM is 42% (32%), while for OSEM+PSF, it is 31% (19%), with (and without) post-filtering, respectively. But with OSEM+PSF+BC, the spill-in contribution from the bladder was relatively low (below 5%, either with or without post-filtering). These results were further validated using the NEMA phantom and patient data for which OSEM+PSF+BC showed about 70–80% spill-in reduction around the bladder edges and increased contrast-to-noise ratio up to 36% compared to OSEM and OSEM+PSF reconstructions without post-filtering.

**Conclusion:**

The spill-in effect is dependent on the activity in the hot region, lesion size and location, as well as post-filtering; and this is more evident in SUV_max_ than SUV_mean_. However, the recently proposed background correction method facilitates stability in quantification and enhances the contrast in lesions with low uptake.

**Electronic supplementary material:**

The online version of this article (10.1186/s40658-018-0233-8) contains supplementary material, which is available to authorized users.

## Background

Positron emission tomography (PET) has established applications in oncology, cardiology and neurology [1–3] using standardised uptake value (SUV) for quantification, diagnosis and post-therapeutic response prediction [4]. However, as PET involves injection of radiotracers, concerns have been raised on the effectiveness of some common tracers in imaging areas with high radiotracer uptake (hot regions) such as the brain, urinary bladder, myocardium and spine [5–7]. These concerns stem from the observation that activity from the hot regions may interfere with PET quantification and visualisation of nearby lesions, tumours and abnormalities. As a result, nearby lesions have their SUVs overestimated, and in some cases, lesions can be totally missed [8]. This effect is often referred to as the “spill-in” or “shine-through” effect [8–10].

For example, in clinical practice, the spill-in effect from the bladder to the surroundings is often addressed using techniques such as bladder voiding prior to acquisition, catheterisation, irrigation and retrograde filling [11]. However, none of these techniques has been proven to be 100% efficient or give reliable results [12–15]. They are also uncomfortable and invasive for the patient [15], whereas PET is meant to be a minimally invasive imaging technique. Due to these challenges, alternative tracers are sometimes used because of their minimal urinary excretion [16–18], but such tracers still have some limitations with regards to patient sensitivity and specificity [19]. These issues clearly suggest the need for a more practical correction technique for the spill-in of activity from hot regions to the surroundings.

Correction of spill-in effect is essential in PET imaging for enhanced quantitative accuracy and better lesion detection [20–23]. This correction can be voxel or region of interest (ROI)-based and implemented either within or after the image reconstruction [24–26]. However, spill-in correction is commonly applied to brain imaging, with little or no studies involving imaging of the pelvic regions. Also, some correction techniques require a priori information about the lesion shape and size; hence, increasing the possibility of erroneous estimation when this information is not precisely known. They might also be computationally demanding [26].

A recent novel simulation study [27] suggested a reconstruction approach to correct for the high physiological radioactive concentration in order to address these aforementioned issues and improve lesion quantification. This method involves segmentation, forward-projection and reconstruction-based correction. However, the performance of this correction method on a state-of-the-art scanner, including accurate iterative scatter correction and point-spread-function (PSF) correction inside the reconstruction, remains to be studied. The previous work used the General Electric (GE) Advance NXi scanner with a spatial resolution of 4.8 mm full width at half maximum (FWHM), whereas the present work used the GE Signa PET/MR scanner with a spatial resolution of 4.3 mm FWHM. Also, the previous work corrected for the scattered and random events before the reconstruction, whereas this current work incorporated all corrections (attenuation, normalisation, scatter and randoms) in the reconstruction, as in the state-of-the-art reconstruction algorithms.

The aim of this study was to implement the recently proposed background correction and evaluate its performance in terms of lesions’ quantification accuracy and contrast, using both simulated and real clinical PET/MR data. The performance was assessed for lesions near a background hot region, as a function of background activity, lesion-to-background distance, lesion size, spatial resolution, and degree of post-filtering.

## Materials and methods

### Datasets and PET/MR system

**Fig. 1:**
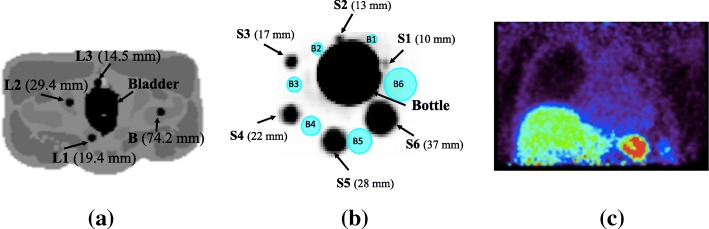
The datasets used for this study: **a** simulation from XCAT2 phantom consisting of the bladder, three lesions (L1–L3) and one background lesion (B) placed at different distances (shown in brackets) from the bladder; **b** NEMA phantom consisting of a hot bottle at the centre, surrounded by six spheres (S1–S6) with diameters shown in brackets. The blue spheres (B1–B6) were used to estimate the background activity for the estimation of contrast-to-noise ratio (CNR); **c** patient data showing high activity in the spleen and liver

Two datasets were primarily used in this study: (i) simulated data from digital XCAT2 phantom [28] and (ii) experimental data from a NEMA IQ phantom [29]. Additionally, a patient abdominal scan was used for further validation. These datasets are shown in Fig. 1.

The experimental studies were acquired using the GE Signa PET/MR scanner at Invicro Ltd., with 25 cm axial field of view (FOV), 60 cm transaxial FOV, 70 cm ring diameter, 25 × 4.0 × 5.3 crystal size in mm, and energy resolution of 10.5% with an energy window 425–650 keV [30]. The simulation was performed using an analytical model of the same scanner.

### Simulations

We simulated the pelvic bed using digital XCAT2 phantom with a typical [^18^F]-fluorodeoxyglucose ([^18^F]-FDG) distribution. Hot bladder was simulated with a fixed volume (500 ml) and various activities (i.e. SUVs 8.5, 19.3, 33.8 and 55.5) allocated as obtained from the literature [31, 32]. This is to mimic the progressive increase in bladder activity due to the radiotracer accumulation during a typical PET examination. Three lesions labelled L1–L3 each with diameter 10 mm and fixed activity value (SUV 8) were placed at different locations around the bladder as shown in Fig. 1a. We also simulated an increase in bladder volume and activity during a typical 90 min scan using extracted values from the literature [33, 34] at time points 1, 10, 30, 45 and 90 min. This was done to investigate the spill-in effect from the bladder to the surrounding lesions as a function of increasing bladder volume, bladder activity and lesion distance from the bladder. Additionally, lesion diameters ranging from 6 mm to 12 mm with a step size of 2 mm were simulated for all bladder SUVs to investigate the spill-in effect as a function of lesion size. For each lesion diameter, we also added same-sized background lesion (B) farther from the bladder such that the background lesion is not affected by the spill-in effect from the bladder. This was done to distinguish between spill-in and spill-out effects as it affects lesion quantification, especially for small lesions. The emission and attenuation images were generated for each of these bladder and lesions combinations.

Fully three-dimensional (3D) analytical simulation was done using the Software for Tomographic Image Reconstruction (STIR) package [35], considering all sinograms with ring differences (RD) less than or equal to 44, and span 1. To approximate the blurring effect from the scanner, the simulated images were first convolved with a Gaussian filter having full width at half maximum (FWHM) as that of GE PET/MR PSF (4.2 mm and 5.7 mm FWHM in transverse and axial planes, respectively [36]) at the forward-projection stage. The forward projector used is based on Siddon’s ray tracing algorithm [37], tracing 10 tangential rays for each bin. Attenuation correction factors were calculated from the attenuation image, and this was used to attenuate the emission sinogram. Constant normalisation and random sinograms were also generated, with the random counts making up to 20% of the total projection data. Scatter was estimated analytically using the single scatter simulation (SSS) approach and scaled to make the scatter count 35% of the total simulated events. The random and scatter sinograms were used to generate the additive term, while the attenuation and normalisation sinograms were used as multiplicative terms. Time of flight (TOF) was not simulated for this study.

Poisson noise was added to the sinograms to simulate 65 × 10^6^ counts, and 20 noise realisations were performed for statistical analysis. Spherical ROIs equal in diameter to the size of the lesions were placed in the position of each lesion in order to extract the uptake values (SUV_mean_ and SUV_max_) for each noise realisation. The mean $\left(\overline{M}\right)$, standard error of the mean (*SEM*) and bias (*B*) from all the 20 realisations (as defined in Eq. 1(i–iv)) were used as figures of merit to show the differences in SUV values for both uncorrected and corrected images.

1.i $$ \overline{M}=\frac{1}{N}{\sum}_{i=1}^N{f}_i $$

1.ii $$ SEM=\frac{SD}{\sqrt{N}} $$

where

1.iii $$ SD=\sqrt{\frac{1}{N-1}{\sum}_{i=1}^N{\left({f}_i-\overline{M}\right)}^2} $$

1.iv $$ B=\frac{\overline{M}-T}{T}\times 100 $$

*f*_*i*_ is the SUV from a single noise realisation, *N* is the total number of noise realisations (= 20) and *T* is the SUV of the true simulated image.

The % change in lesion (*l*) SUV (*∆SUV*_*l*_) as the bladder SUV increases from SUV 8.5 to SUV 55.5 was also estimated using:

2 $$ {\Delta  SUV}_l\left(\%\right)=\frac{SUV_{l(55.5)}-{SUV}_{l(8.5)}}{SUV_{l(8.5)}}\times 100 $$

where *SUV*_*l*(8.5)_ and *SUV*_*l*(55.5)_ represent the lesion SUV at bladder SUVs 8.5 and 55.5, respectively.

To further investigate the spill-in effect from the bladder to the surroundings, we created shells of different voxels (from 2 to 10 voxels with a step size of 2 voxels) around the bladder by performing a morphological operation on the bladder mask to obtain the edge mask:

3 $$ \mathrm{edge}\ \mathrm{mask}=\mathrm{dilation}\left(\mathrm{bladder}\ \mathrm{mask},n\right)-\mathrm{bladder}\ \mathrm{mask} $$

where *dilation*(*bladder mask*, *n*) means dilating the bladder mask by *n* voxels using a sphere structuring element in MATLAB.

**Fig. 2:**
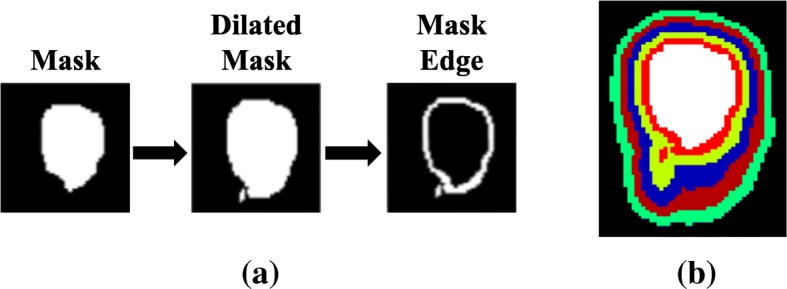
The schematic 2D representation of the technique used to extract the voxel values around the edges of the simulated bladder. **a** represents the procedure for extracting the voxel values, while **b** shows a 2D representation of all the dilated 3D regions around the bladder (i.e. the white region) from 2 voxels to 10 voxels with a step size of 2 voxels

The resulting edge mask was then used to extract the uptake values around the bladder in both true simulated and reconstructed mean images as illustrated in Fig. 2a.

### Validation by real data

#### Experimental phantom

A phantom experiment was performed with the GE Signa PET/MR scanner at Invicro Ltd. using the NEMA image quality (IQ) phantom without the wall (Fig. 1b) [29]. This phantom consisted of six fillable spherical spheres S1 to S6 (with diameters 10, 13, 17, 22, 28 and 37 mm, respectively, and filled with 5.38 MBq of [^18^F]-FDG). A high-activity 500 mL bottle (filled with 77.9 MBq of [^18^F]-FDG) was placed at the centre of the phantom. The experimental set-up involved a simultaneous PET/MR acquisition with a 5-min static PET acquisition and 2-point Dixon MR 3D acquisition. List of events and singles rates for crystals were extracted from the listmode file from the scanner, and these were converted to emission and random sinograms using STIR utility. The normalisation file was also extracted from the scanner and was converted to the normalisation sinogram using STIR. Attenuation factors were obtained from the in-phase MRAC image, and scatter was estimated using STIR.

The contrast-to-noise ratio (CNR) was used to evaluate the signal quality and noise properties of each reconstruction algorithm. This was done by estimating the mean activity and standard deviation in each sphere, as well as in pre-selected background spheres. The background spheres were represented as six spherical ROIs placed around the hot bottle which were exactly of the same size as the spheres and located at approximately the same distance from the hot bottle (as shown in Fig. 1b).

So, the CNR was estimated for each sphere (*i* = 1, 2, …, 6) using:

4 $$ {\mathrm{CNR}}_{\mathrm{sphere}(i)}=\frac{{\mathrm{Activity}}_{\mathrm{Sphere}(i)}-{\mathrm{Activity}}_{\mathrm{Background}(i)}}{\sqrt{{\mathrm{SD}}_{\mathrm{Sphere}(i)}^2+{\mathrm{SD}}_{\mathrm{Background}(i)}^2}} $$

Also, the spill-in activity from the bottle to the surroundings was estimated using the same morphological operation as in the simulation studies (shown in Eq. 3).

#### Patient data

For further validation, we used patient data (Fig. 1c) acquired with the GE PET/MR scanner at Invicro Ltd. This was acquired during a lung fibrosis examination using an [^18^F]-based radiotracer. This data did not exhibit high activity in the bladder, but in the spleen and the liver. Thus, for demonstration purposes, we chose the spleen as the background hot region. We reconstructed the patient data using the same settings as for the phantom experiment. We also carried out the morphological dilation operation (in Eq. 3) on all reconstructed images so as to estimate the spill-in effect around the hot region (spleen).

### Data reconstruction and spill-in correction

Image reconstruction was done using the 3D iterative ordered subset expectation maximisation (OSEM) algorithm in STIR library. Attenuation, normalisation, random and scatter corrections were performed by including the multiplicative and additive terms in the reconstruction algorithm. For the simulation, 28 subsets and 30 full iterations were used to ensure reasonable convergence of the recently proposed correction algorithm. Meanwhile, for the real data, fully 3D reconstruction with mixed span factors according to GE’s configuration (span 1 for |RD| > 1; and span 3 for RD = − 1,0,1) was done using OSEM with 28 subsets and 20 full iterations, incorporating all corrections within the reconstruction. The reconstructed images have 256 × 256 × 89 voxels with size 2.34 × 2.34 × 2.78 mm^3^, and they were post-filtered with a 4-mm isotropic 3D Gaussian filter.

Spill-in correction was done using the two techniques as outlined below:

- PSF reconstruction

This involves incorporating a spatially invariant PSF into the OSEM reconstruction in both forward and backward projections as illustrated in Eq. (5). This PSF was specified as a 3D Gaussian filter with 4.2 mm and 5.7 mm FWHM in transverse and axial planes, respectively, according to experimental values obtained for the GE PET/MR scanner [36].

5 $$ {H}_{ij}^{\prime }={\sum}_k{H}_{kj}{PSF}_{ik} $$

where *H*_*kj*_ represents the system matrix and ${H}_{ij}^{\prime }$ is the system matrix convolved with the system PSF in both forward and back projections [38, 39]. This reconstruction method is referred to as OSEM+PSF in this study.

(b) Background correction (BC)

**Fig. 3:**
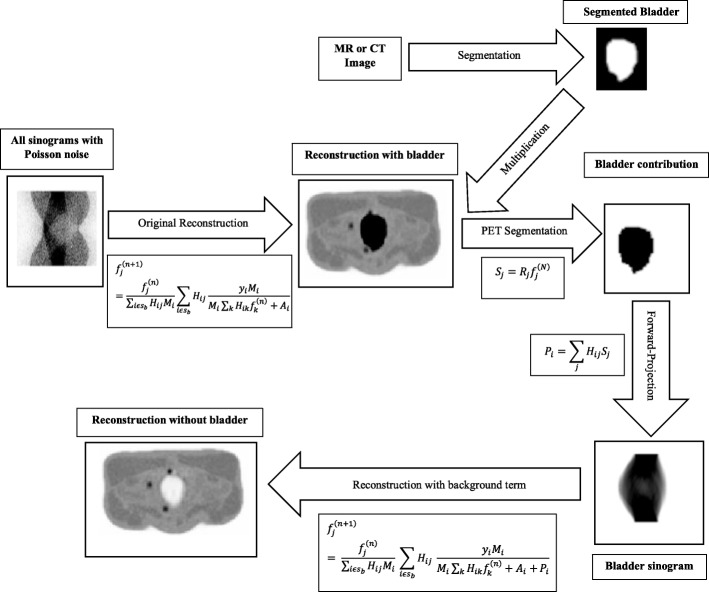
Schematic description of the background correction technique, as demonstrated for the XCAT2 phantom simulation. In this study, the bladder was automatically segmented from the XCAT2 phantom, but in principle, the hot region can be segmented from either the CT or MR image

For the simulation, the bladder (hot region) was automatically segmented from the XCAT2 phantom to obtain the bladder mask *S*_*j*_. This was then multiplied by the OSEM+PSF reconstructed image ${f}_j^{(N)}$ (taken at five iterations) to obtain the bladder contribution in the image (i.e. ${B}_j={S}_j{f}_j^{(N)}\Big)$, which was then forward-projected using the same projection matrix for the simulation (${P}_i={\sum}_j{H}_{ij}^{\prime }{B}_j\Big)$ and included in the reconstruction as a background term along with the additive term (as shown in Eq. 6). For the real data, the highly radioactive bottle that accompanied the NEMA phantom and the hot spleen of the patient were segmented from the Dixon in-phase MRAC image before forward-projecting it to obtain the background contribution.

6 $$ {f}_j^{\left(n+1\right)}=\frac{f_j^{(n)}}{\sum_{i\in {J}_j}{H}_{ij}^{\prime }{M}_i}\ {\sum}_{i\in {J}_j}{H}_{ij}^{\prime}\frac{y_i\ {M}_i}{M_i{\sum}_{k\in {I}_i}{H}_{ik}^{\prime }{f}_k^{(n)}+{A}_i+{P}_i} $$

where ${f}_j^{(n)}$ is the uncorrected image, *y*_*i*_ is the emission sinogram, ${H}_{ij}^{\prime }$ is the system matrix with the PSF, *M*_*i*_ is the multiplicative term, *A*_*i*_ is the additive term, *P*_*i*_ is the bladder background term and ${f}_j^{\left(n+1\right)}$ is the final corrected image without the bladder contribution. The flow sequence is shown in Fig. 3. Since this technique also includes OSEM+PSF, it will be referred to as OSEM+PSF+BC in this study.

## Results

### Simulations

#### Investigating the spill-in effect as a function of bladder activity, lesion size and post-filter using OSEM reconstruction

**Fig. 4:**
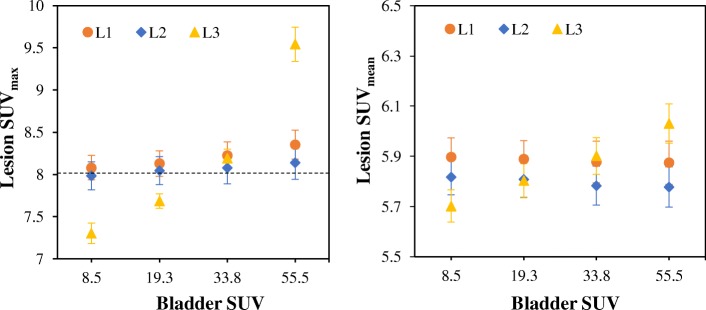
The lesion uptake as the bladder SUV increases for images reconstructed with OSEM. These are the mean SUVs from all the 20 noise realisations and for lesion diameter 10 mm at 30 full iterations. The error bars are the standard error of the mean (SEM) while the dashed horizontal line denotes the true simulated lesion SUV

Figure 4 shows the mean lesion SUV_max_ and SUV_mean_ for OSEM reconstruction at 30 full iterations. As the bladder SUV increases, the lesion SUV_max_ also increases for all lesions, with the highest SUV in L3. However, SUV_mean_ increases only for L3. The % bias and the % change in lesion SUV as the bladder SUV increases from SUV 8.5 to SUV 55.5 (using Eq. 2) are presented in Table 1.

**Table 1:** The bias and relative change in lesion SUV_max_ and SUV_mean_ for all the simulated bladder SUVs and for lesion diameter 10 mm. These are estimated from the mean SUVs from all the noise realisations. (The % bias values are given in parentheses)

Bladder SUV	Simulated lesions					
	L1		L2		L3	
	SUV_max_	SUV_mean_	SUV_max_	SUV_mean_	SUV_max_	SUV_mean_
SUV 8.5	8.08 (1.00)	5.90 (− 26.28)	7.98 (− 0.21)	5.82 (− 27.27)	7.30 (− 8.72)	5.70 (− 28.72)
SUV 19.3	8.13 (1.61)	5.89 (− 26.39)	8.05 (0.57)	5.81 (− 27.39)	7.68 (− 3.95)	5.80 (− 27.47)
SUV 33.8	8.23 (2.82)	5.88 (− 26.53)	8.08 (0.97)	5.78 (− 27.71)	8.19 (2.41)	5.90 (− 26.23)
SUV 55.5	8.35 (4.41)	5.87 (− 26.57)	8.14 (1.78)	5.78 (− 27.78)	9.54 (19.27)	6.03 (− 24.61)
% Change	3.37	− 0.39	1.99	− 0.70	30.67	5.77

For lesion L3, the % bias in SUV increases as the bladder SUV increases, and this is more pronounced for SUV_max_ than SUV_mean_. However, for lesions L1 and L2, only SUV_max_ has an increased % bias as the bladder SUV increases, SUV_mean_ seems constant. At bladder SUV 55.5, SUV_max_ of lesion L3 (closest to the bladder) has a % bias of 19.3% while for L2 (further away), the % bias in SUV_max_ is only 1.8%. As bladder SUV increases from 8.5 to 55.5, the % SUV change for L3 is 30.67% and 5.77% for SUV_max_ and SUV_mean_, respectively, while the % change in SUV_max_ is less than 4% for lesions L1 and L2.

**Fig. 5:**
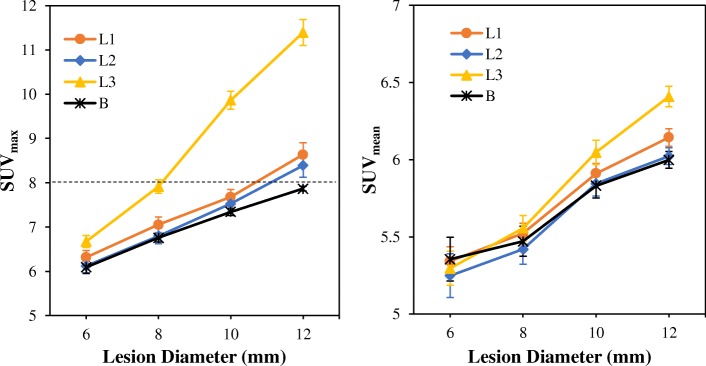
The spill-in and spill-out effects as a function of lesion size for lesions L1–L3 and background lesion B at bladder SUV 55.5. The SUVs were obtained from OSEM reconstructed images using the mean SUVs of all noise realisations at 30 full iterations with a 4-mm Gaussian post-filter. The error bars are the standard error of the mean (SEM) while the dashed horizontal line denotes the true simulated lesion SUV. Background lesion B is the reference which shows the expected lesion SUV without spill-in effect

Figure 5 shows the spill-in and spill-out effects on lesion quantification as a function of lesion size. This is shown for lesions L1–L3 and background lesion B at bladder SUV 55.5. As expected, the spill-out effect from the lesions to the colder background causes an underestimation in lesion SUV for small diameter lesions, but the SUV increases as the diameter increases. However, the lesion SUV is further influenced by spill-in effect from the bladder to the lesions, and this depends on the lesion distance. Lesion L3, which is the closest lesion to the bladder, has the highest SUVs while lesion L2, which is farther away, has the least.

#### Effect of correction techniques on reduction of spill-in effect

**Fig. 6:**
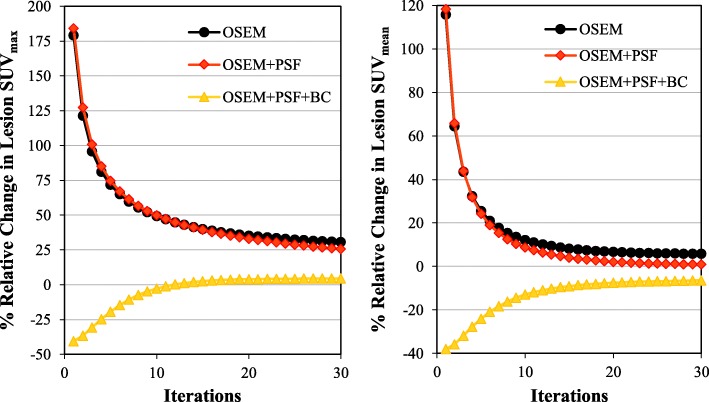
The % relative change in lesion SUV (as bladder SUV increases from SUV 8.5 to SUV 55.5) as a function of increasing iteration. This is obtained from a single noise realisation and for lesion L3 of diameter 10 mm with a 4-mm Gaussian post-filter

Figure 6 shows how the lesion SUV changes iteratively for all reconstruction techniques as bladder SUV increases from SUV 8.5 to SUV 55.5. This shows that % change in lesion SUV reduces as iteration increases. At 30 iterations, the % change in lesion SUV_max_ is about 31%, 26% and 4% for OSEM, OSEM+PSF and OSEM+PSF+BC, respectively, while for lesion SUV_mean_, it is about 6%, 1% and − 6% for OSEM, OSEM+PSF and OSEM+PSF+BC, respectively.

Table 2 shows the estimated spill-in activity from the bladder to the surrounding regions for both uncorrected and corrected images at 30 full iterations with and without post-filtering. This is the percentage activity difference in the dilated shells between the true simulated image and the reconstructed image. There is a large spill-in activity within 2 voxels around the bladder as the % activity difference between the simulated and reconstructed images is 31.5% (41.8%) for OSEM and 18.7% (30.8%) for OSEM+PSF reconstructions without and with post-filtering, respectively. This shows that filtered images show increased spill-in activity of about 33% and 65% for OSEM and OSEM+PSF respectively, compared with the unfiltered images. At 4 voxels around the bladder, the activity difference around the bladder dropped greatly to 9.3% (8.5%) and 2.6% (2.7%), and at 10 voxels from the bladder, the activity difference is 2.8% (3.4%) and 0.6% (1.5%) for OSEM and OSEM+PSF, respectively. However, with the correction technique, the % activity difference is within 0.5% and 4.7% with and without post-filtering. It could be observed that OSEM has the highest upper limit of agreement (LOA), compared to OSEM+PSF and OSEM+PSF+BC. Also, the upper LOA for all methods reduces as we move voxels away from the bladder.

**Table 2:** Estimation of the spill-in activity from the bladder to the surrounding regions. This is estimated from both filtered and unfiltered mean images with simulated bladder SUV 55.5 at 30 iterations (the filtered results are given in parentheses). $\left(\overline{d}\right)$ is the difference between the mean values of the true simulated and reconstructed images for all voxels in the dilated region. LOA is the 95% Limit of Agreement of $\left(\overline{d}\right)$, with ± showing the upper and lower limits

Dilated regions (voxels)		OSEM	OSEM+PSF	OSEM+PSF+BC
1–2	% Difference $\left(\overline{d}\right)$	31.5 (41.8)	18.7 (30.8)	3.3 (1.6)
	SD^1^	26.4 (28.4)	25.0 (30.2)	15.4 (11.7)
	LOA^2^	− 20.3 to + 83.3 (− 13.9 to + 97.6)	− 30.4 to + 67.8 (− 28.4 to + 89.9)	− 26.9 to + 33.5 (− 21.4 to + 24.6)
3–4	% Difference $\left(\overline{d}\right)$	9.3 (8.5)	2.6 (2.7)	4.7 (3.9)
	SD	10.6 (9.5)	8.0 (8.9)	10.0 (8.8)
	LOA	− 11.4 to + 30.0 (− 10.1 to + 27.0)	− 13.1 to + 18.4 (− 14.7 to + 20.1)	− 15.0 to + 24.3 (− 13.4 to + 21.2)
5–6	% Difference $\left(\overline{d}\right)$	5.4 (6.4)	2.4 (3.6)	1.3 (2.5)
	SD	12.9 (7.8)	8.7 (6.7)	10.9 (8.3)
	LOA	− 19.9 to + 30.7 (− 8.8 to + 21.6)	− 14.8 to + 19.5 (− 9.5 to + 16.7)	− 20.0 to + 22.7 (− 13.8 to + 18.8)
7–8	% Difference $\left(\overline{d}\right)$	4.0 (4.3)	1.6 (2.0)	1.4 (1.6)
	SD	12.8 (7.9)	8.9 (7.1)	11.2 (8.3)
	LOA	− 21.1 to + 29.9 (− 11.2 to + 19.8)	− 16.0 to + 19.1 (− 12.0 to + 15.9)	− 20.4 to + 23.3 (− 14.6 to + 17.9)
9–10	% Difference $\left(\overline{d}\right)$	2.8 (3.4)	0.6 (1.5)	0.5 (1.3)
	SD	11.6 (7.0)	9.1 (6.4)	10.7 (7.4)
	LOA	− 19.9 to + 25.5 (− 10.2 to + 17.1)	− 17.2 to + 18.4 (− 11.0 to + 14.1)	− 20.4 to + 21. 3 (− 13.1 to + 15.8)

^1^$SD=\sqrt{\frac{1}{n-1}\sum \limits_{k=1}^n{\left({d}_k-\overline{d}\right)}^2}$, *k* represents each voxel in the dilated region

^2^ $$ LOA=\overline{d}\pm 1.96 SD $$

**Fig. 7:**
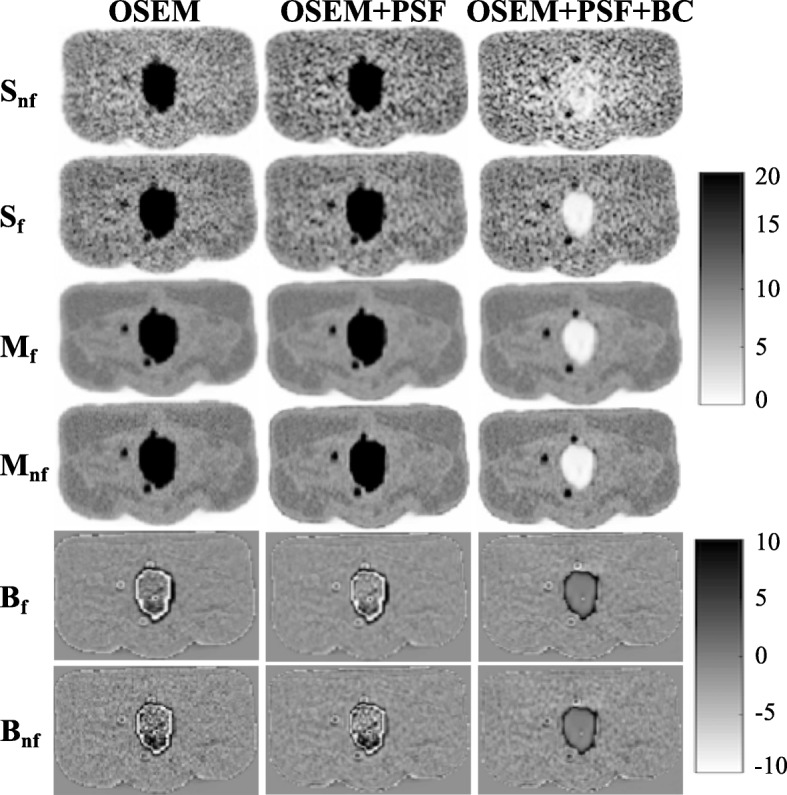
Images showing improvement in lesion detection and reduction of bias around bladder edges with the correction technique. This is shown for bladder SUV 55.5 and lesion diameter 10 mm at 20 full iterations. *S*_f_ and *S*_nf_ are the single noise realisation images with and without filtering, respectively, while *M*_f_ and *M*_nf_ are the mean images from 20 noise realisations with and without filtering respectively. *B*_f_ and *B*_nf_ are the bias images (i.e. difference between the mean image and the true simulated image) with and without filtering respectively. The bias image in OSEM+PSF+BC was estimated by first removing the bladder from the true simulated image

This spill-in activity can also be seen around the bladder edges in the uncorrected images (as shown in Fig. 7). This causes a bias around the bladder edges making the bladder appear bigger, hence affecting the visibility of nearby lesions.

#### Dependence of the background correction method on segmentation accuracy

**Fig. 8:**
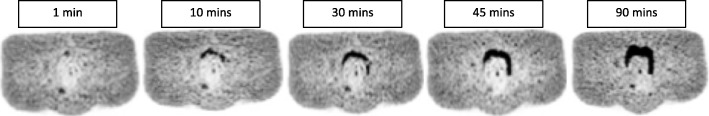
Demonstration of how inaccurate segmentation can limit the accuracy of the background correction technique. This is shown for a single noise realisation, with a 4-mm post-filter. All images are displayed with the same maximum SUV threshold value 20

To demonstrate the dependence of the recently proposed background correction method on segmentation accuracy, we simulated an increase in bladder volume and activity during a 90-min scan typical of PET/MR scan at time points 1, 10, 30, 45 and 90 min. Each image was reconstructed with background correction (OSEM+PSF+BC), but the segmented bladder mask at 1 min timepoint was used for the correction in all cases. The resulting background corrected images are displayed in Fig. 8.

This result shows that bladder expands majorly at the anterior superior end towards the rectum and the prostate. The error in segmentation is pronounced after 10 mins.

### Validation by phantom experiment and patient data

**Fig. 9:**
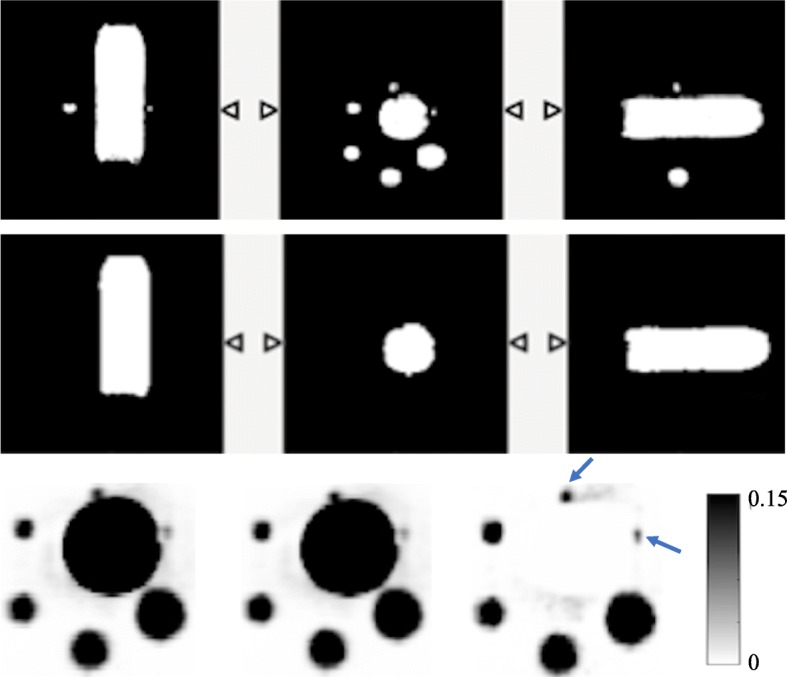
The NEMA bottle phantom used for the validation. The first row shows the MRAC image of the phantom and the middle row is the segmented bottle from the MRAC image, while the last row is showing the coronal view of the reconstructed images at three full iterations with a 4-mm Gaussian filter for OSEM, OSEM+PSF and OSEM+PSF+BC reconstructions, respectively. The blue arrows in the reconstructed images are pointing to the spheres in which there is visual improvement

Figure 9 shows the MRAC, segmented bottle and reconstructed images of the bottle phantom. OSEM+PSF+BC image shows improved visibility especially for S1 and S2.

**Fig. 10:**
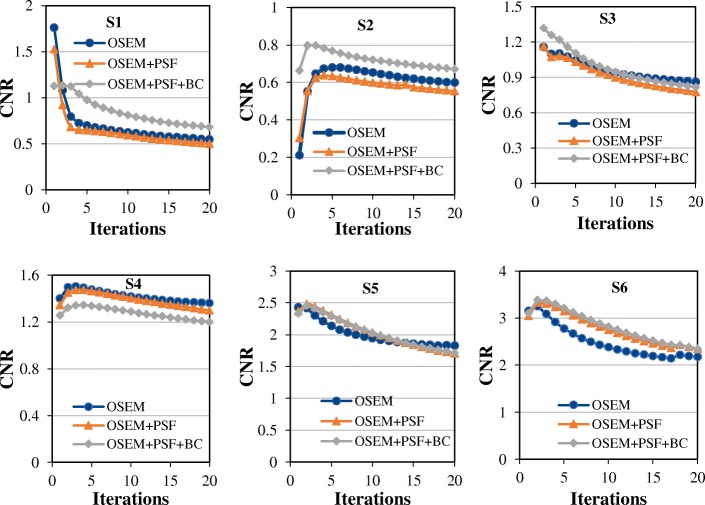
Plots of the CNR of the NEMA phantom spheres S1–S6 against the iteration for all the reconstruction techniques across the 20 full iterations and without post-filtering. The CNR was estimated using the mean activity values in the spheres and background

Figure 10 shows the CNR of all the spheres as a function of iteration. As expected, the CNR increases as the sphere diameter increases, but reduces with the number of iterations. OSEM and OSEM+PSF have almost the same CNR for all spheres, while OSEM+PSF+BC shows higher CNR than OSEM and OSEM+PSF for the two smallest spheres, and the ones closest to the bottle (S1 and S2). OSEM+PSF+BC does not show any clear improvement over OSEM and OSEM+PSF for larger spheres (S3–S6).

**Fig. 11:**
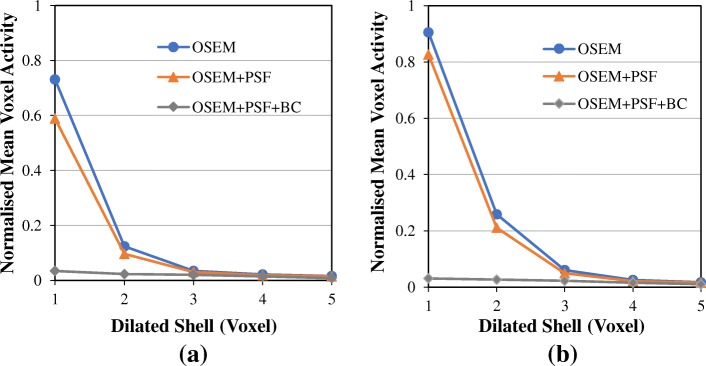
The normalised mean activities within the dilated shells surrounding the NEMA bottle obtained at three full iterations with **a** no post-filter and **b** 4-mm Gaussian post-filter. This demonstrates how spill-in activity from the hot bottle to the surrounding shells reduces as we move farther away from the bottle. The voxel activities were normalised with the actual activity in the spheres

Figure 11 shows the normalised mean activity in each of the dilated shell surrounding the hot NEMA bottle. Although the surrounding shells should have zero activity, the reconstructed images show a considerable amount of spill-in activity from the hot bottle to the surrounding shells. Within 1 voxel around the hot region, the activity is about 75% and 90% of the activity in the sphere for OSEM; and 60% and 80% for OSEM+PSF, with and without post-filtering respectively. However, at 4 voxels, this activity is greatly reduced to just about 2% for OSEM and OSEM+PSF, either with or without post-filtering. However, for OSEM+PSF+BC, the activity is less than 3% in all cases, showing an improvement of more than 80% and 70% over OSEM and OSEM+PSF reconstructions respectively.

**Fig. 12:**
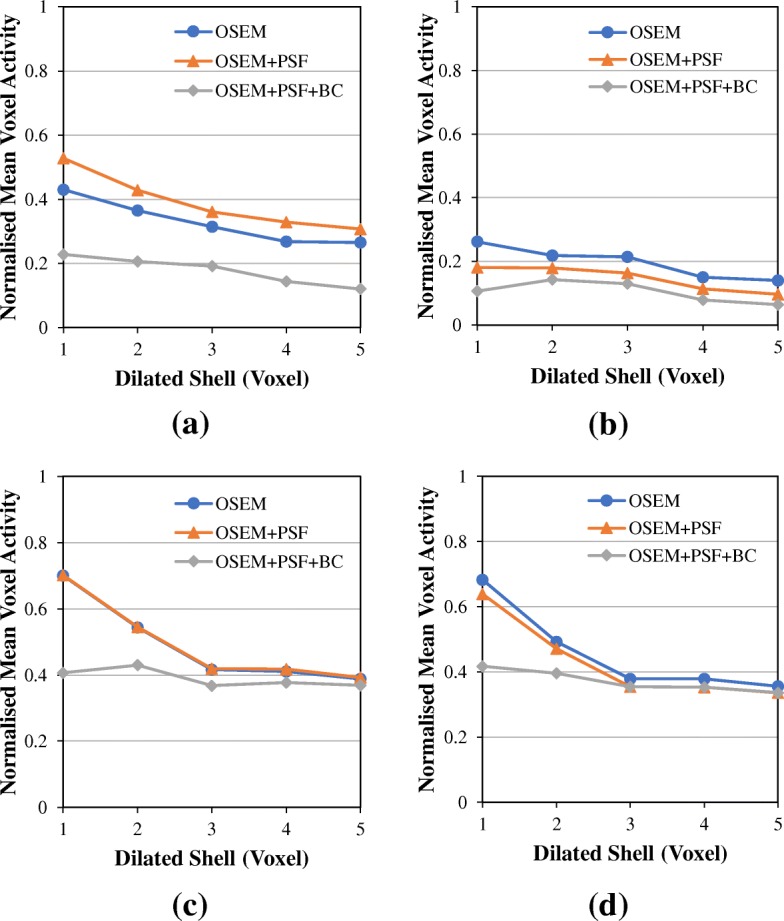
Validating the effect and correction of spill-in effect using patient data. This figure shows the normalised mean values within the dilated shells surrounding the spleen at **a** 3 full iterations with no post-filter, **b** 20 full iterations with no post-filter, **c** 3 full iterations with a 4-mm Gaussian post-filter and **d** 20 full iterations with a 4-mm Gaussian post-filter. The mean activity values in the dilated shells were normalised with the mean activity in the liver

For the patient data, Fig. 12 shows the normalised mean activity in the dilated shells around the spleen in all reconstructed images at 3 and 20 full iterations with and without post-filter. As expected, the mean activity in the dilated shells reduces as we move voxels away from the spleen, with the highest value at 1 voxel from the spleen in all cases. Also, increasing the number of iterations tends to reduce the activity in the dilated shell. For OSEM at voxel 1 and without post-filter, the mean activity is about 43% at three iterations but reduced to only 26% at iteration 20. However, with the application of post-filter, there is no pronounced difference in the voxel activities at 3 and 20 iterations. Also, OSEM+PSF has similar or even higher voxel activity than OSEM at three iterations with and without post-filter, but at 20 iterations, OSEM+PSF shows reduced voxel activity than OSEM. But in all cases, OSEM+PSF+BC has lower and almost constant voxel activity.

## Discussion

We have carried out an extensive investigation with simulated and experimental phantoms on the full suppression of the spill-in effect from background hot regions to the surrounding lesions as a function of increasing background activity, lesion size, and distance to the background, using a recently proposed background correction technique [27].

The simulation results show that lesion uptake increases as bladder activity increases (Fig. 4), and this could be attributed to bladder accumulation, thereby causing spill-in effects [8]. L3 closest to the bladder has the highest change in SUV, while L1 and L2 further away have less SUV variation. This indicates that there is a high probability of SUV overestimation in lesions close to the bladder, and hence, they suffer greatly from the spill-in effect [33, 40]. A similar experiment was conducted by Liu [8] and he concluded that if the lesions are within 40–50 mm from the hot source, the estimated SUV values are overestimated and therefore invalid. However, for our study, this SUV overestimation is only experienced in lesions within 15–20 mm from the bladder. This improved result may be due to the improved resolution of the simulated GE PET/MR scanner, which makes the effect less prominent (as seen in Additional file 1: Figure S4).

We also discovered that the spill-in effect seems to increase with lesion size, and this is more evident in proximal lesions to the bladder (Fig. 5). As expected, spill-out effect from the lesions to the background causes an underestimation in lesion SUV for small diameter lesions, but the SUV increases as the diameter increases. However, the lesion SUV is further influenced by spill-in effect from the bladder to the lesions, and this is dependent on the lesion distance. Lesion L3 which is the closest lesion to the bladder has the highest bias in SUVs while the background lesion has the least. For lesion L3, the spill-in effect seems to increase with lesion diameter. This is because smaller lesions suffer from both spill-out effect to the colder region and spill-in effect from the hot background region. These two effects might sometimes cancel out, thereby giving a false impression of quantification accuracy in smaller lesions (as depicted in our study). However, for bigger lesions close to the bladder, spill-in effect is the major effect, thereby leading to a pronounced overestimation in SUV. For lesions L1 and L2, however, the impact of spill-in effect on SUV quantification is small, and only evident for SUV_max_ (details in Additional file 1: Figure S2). This also re-iterates the strong dependence of spill-in effect on lesion distance.

It is worthy of note that the spill-in effect is more pronounced in lesion SUV_max_ than SUV_mean_ as demonstrated by the results in Figs. 4, 5 and 6. This implies that if one is interested in quantification using SUV_mean_, then, the spill-in effect is minimal and can potentially be ignored. However, the fact that SUV_max_ is the global clinical standard for quantification informs the need for proper spill-in correction. Our study also shows that SUV overestimation as a result of spill-in effect reduces over iteration (as seen in Figs. 6 and 12, and also Additional file 1: Figures S1, S5 and S6). Therefore, in order to reduce the spill-in effect, slightly increasing the number of iteration might be a good alternative. However, this will be at the expense of reduced CNR and increased noise.

OSEM+PSF and OSEM+PSF+BC were used as correction techniques for the spill-in effect (their convergence properties are shown in Additional file 1: Figure S1). For L3, there does not seem to be any major improvement in OSEM+PSF reconstruction over OSEM especially for SUV_max_ (as seen in Fig. 6). This could be because of the closeness of the lesion to the bladder, coupled with the blurring effect caused by post-filtering. This seems to nullify the recovery already obtained by incorporating PSF into the reconstruction algorithm, thereby making both ordinary OSEM and OSEM+PSF reconstructions to behave in a similar way. This blurring effect could further be confirmed by a pronounced bias at the bladder edges due to the spill-in activity from the bladder as shown in Fig. 7, as well as the edge analysis in Table 2. OSEM and OSEM+PSF are greatly affected by filtering, but OSEM+PSF+BC is more robust and less sensitive to filters. Another reason for the similar behaviour between OSEM and OSEM+PSF might be the simplistic spatially invariant resolution modelling in this study. This simple resolution modelling has been shown by past studies to increase edge artefact and system blurring [41].

From the NEMA phantom experiment (Fig. 11), this spill-in activity from the bottle can cause between 20 and 90% activity overestimation in a sphere if it is within 1–2 voxels away from the hot bottle, but it reduces as we move further away from the bottle. The spill-in activity reduced greatly with OSEM+PSF+BC as it shows a value less than 3% in all cases. In this phantom experiment, spill-in effect is only prominent within 3 voxels (less than 10 mm) around the hot bottle, whereas in the simulation study, this effect could potentially extend to about 15 mm with a very high activity in the bladder. This is an indication that this spill-in effect is strongly dependent on the activity in the hot region. Further validation of this technique using patient data also establishes the spill-in effect as a function of distance from the hot region (Fig. 12). OSEM and OSEM+PSF images show an increased activity value in the immediate vicinity of the spleen compared to OSEM+PSF+BC image, but this disparity in activity value decreases as we move further away from the spleen.

From the results shown in Figs. 6, 7 and 9, it could be seen that better lesion detection and quantification could be achieved with the recently proposed background correction technique, thereby potentially enhancing low contrast lesion detectability and better diagnosis. This was also confirmed by the NEMA phantom (Fig. 10) as OSEM+PSF+BC demonstrate higher CNR than OSEM and OSEM+PSF especially at lower iterations and for smaller spheres. Also, the recently proposed background correction method has a stable performance for both lesion SUV_mean_ and SUV_max_ (demonstrated by almost 0% change in Fig. 6) irrespective of the bladder activity and application of post-filter. This performance stability is an indication that the correction method can be used in the clinic for treatment response monitoring; however, this is still subject to further validation.

When compared with previously published results [8, 27], our current study showed clearer and better results demonstrated on acquired PET data probably due to improved correction technique, incorporation of PSF in the reconstruction, and improved scanner resolution. There is an indication that the recently proposed background correction technique is robust and efficient in removing spill-in activity from the high-activity regions to the surroundings, thereby producing more reliable lesion quantification and better lesion visibility, compared with other correction techniques. Moreover, it is less tedious in that there is no need to calculate each lesion activity separately.

However, the recently proposed background correction technique is highly dependent on segmentation accuracy as shown in Fig. 8. This is important especially for pelvic scans where the bladder changes in volume and shape over time. Our result showed that the segmentation error is pronounced after 10 mins where the bladder expands majorly in an anterior direction towards the rectum and superior to the pelvic organs. This is in line with past studies [42, 43] which showed that bladder expands primarily in the superior anterior direction, and hence, addition of anisotropic margins to the bladder is necessary. The translation of this correction technique to clinical application would need to take into account the inaccuracy in bladder segmentation either by adding additional margins or by manual correction as suggested by past studies.

With the emergence of new tracers such as ^68^Ga-prostate-specific membrane antigen (^68^Ga-PSMA) for prostate imaging, this correction technique can also be applicable for correcting shine-through effect in PET/MR imaging which causes loss of resolution and image artefacts due to its significant urinary excretion [19, 40]. Therefore, accurate correction will enhance reliable quantification of PET images and may lead to a tangible breakthrough in ^68^Ga-PSMA imaging.

### Limitations of the study

A major limitation of this work is that there is no TOF implementation, whereas the modelled GE scanner supports TOF. Although TOF has been shown to mitigate errors due to data inconsistency [44], it is not yet certain if TOF implementation can sufficiently correct for spill-in effect, especially for proximal lesions (see TOF reconstructions from GE PET Toolbox in Additional file 1: Figures S5 and S6). However, work is currently ongoing on implementing this in the STIR library, and we therefore aim to include this in our future investigations. Another limitation is the lack of detailed realistic simulations to model some effects such as positron range, non-collinearity, detector response, inter-crystal scattering etc. Furthermore, a crucial approximation is that we modelled an image-based system blurring and used the same analytical model to simulate and reconstruct the data. Although this is helpful for investigating the performance of the algorithm, it would have been preferable to utilise Monte Carlo simulators. Nevertheless, these limitations do not cancel the relevance of the background correction method because we also utilised acquired phantom and patient datasets which demonstrated that the recently proposed background correction method successfully corrects for the background activity.

Also, we used a spatially invariant model of the PSF in this work due to the complexities and computational demands associated with the use of a spatially variant model. Previous works have reported similar behaviour between the two models in terms of resolution, contrast and noise metrics [41, 45]; however, we realise that a spatially variant model of the PSF is more accurate in reducing edge artefact and system blurring. We therefore aim to implement a realistic, spatially variant PSF modelling in our future work, as this might potentially help reduce both the spill-in and spill-out effects.

Clinical translation of this technique would need to place careful emphasis on the segmentation of the high-activity region. For example, it could be possible to use an MR image to segment the region as done in this study. Although segmentation could be performed on CT images as well, this is more challenging as PET/CT acquisitions are not performed simultaneously and there is the potential of bladder expansion between CT and PET scanning due to physiological motion. This has been reported to cause conspicuous distortions in lesions’ shape and location. Another segmentation mismatch could also result from increase in bladder shape and size during a typical PET/MR scanning session. These issues could cause loss of resolution and inadequate quantification and also limit the applicability of this correction approach, as demonstrated in Fig. 8. A potential way of dealing with the segmentation inaccuracies might be to perform segmentation using multi-MR sequences, which could track the bladder change in shape and volume during a typical PET/MR scan. We therefore aim to incorporate appropriate corrections for bladder expansion and motion in our algorithm.

## Conclusion

The effect of increasing activity from hot regions on adjacent lesion quantification, as well as the improvement brought about by the recently proposed background correction technique has been extensively studied in this work. This study shows that lesions relatively close to hot regions (within 15–20 mm) are greatly affected by the spill-in effect, causing reduced visibility and activity overestimation of lesions. This effect is more pronounced in SUV_max_ than SUV_mean_ and reduces over iteration, but it is further aggravated by the use of filter. However, improved quantification and better lesion detectability were achieved with the recently proposed background correction technique irrespective of the lesion size, lesion distance from the hot region, the activity in the hot region or application of post-filter.

We could therefore conclude that the recently proposed background correction method is appropriate for reliable quantification and diagnosis of lesions near a hot region. This is particularly important when examining the pelvic areas for infection, metastases and cancer. Furthermore, this correction technique is not limited to pelvic imaging. It could potentially be applied to imaging of any high-activity region such as the brain, head and neck, myocardium, and bone.

## Additional file

Additional file 1: **Figure S1.** Convergence plots of all the reconstruction algorithms using lesion SUV_max_ and SUV_mean_. **Figure S2.** The variation of the lesion uptake with lesion size and bladder SUV. **Figure S3.** The dependence of spill-in effect on post-filter. This was done with a single noise realisation at 30 iterations. **Figure S4.** The spill-in activity from the bladder to the surroundings as a function of the system resolution (in FWHM), using Eq. 2. **Figure S5.** The normalised mean values within the dilated shells surrounding the NEMA bottle obtained at with (a) 3 iterations and (b) 20 iterations, with non-TOF and TOF reconstructions. **Figure S6.** Normalised mean values within the dilated shells surrounding the spleen obtained with non-TOF and TOF reconstructions at (a) 3 iterations and (b) 20 iterations. **Table S1.** This shows how bladder SUV affects lesion quantification for each lesion diameter. This is expressed as the % change in lesion SUV as bladder SUV increases. The SUV values are the mean values from all noise realisations at 30 full iterations with 4 mm post-filter. (DOCX 139 kb)

## Abbreviations

^68^Ga-PSMA: Prostate-specific membrane antigen labelled with ^68^Ga
BC: Background Correction
CNR: Contrast-to-noise ratio
FOV: Field of view
FWHM: Full width at half maximum
GE: General Electric
IQ: Image quality
OSEM: Ordered subset expectation maximisation
PET: Positron emission tomography
PSF: Point-spread function
RD: Ring difference
ROI: Region of interest
STIR: Software for Tomographic Image Reconstruction
SUV: Standardised uptake values
