# Supplementary material for: Validation of the physiological background correction method for the suppression of the spill-in effect near highly radioactive regions in positron emission tomography
Source: EJNMMI Phys. 2018 Dec 5;5:34. doi: 10.1186/s40658-018-0233-8 (PMC6281548; doi:10.1186/s40658-018-0233-8)
Supplement: Supplementary file 1 — Figure S1. Convergence plots of all the reconstruction algorithms using lesion SUVmax and SUVmean. Figure S2. The variation of the lesion uptake with lesion size and bladder SUV. Figure S3. The dependence of spill-in effect on post-filter. This was done with a single noise realisation at 30 iterations. Figure S4. The spill-in activity from the bladder to the surroundings as a function of the system resolution (in FWHM), using Eq. 2. Figure S5. The normalised mean values within the dilated shells surrounding the NEMA bottle obtained at with (a) 3 iterations and (b) 20 iterations, with non-TOF and TOF reconstructions. Figure S6. Normalised mean values within the dilated shells surrounding the spleen obtained with non-TOF and TOF reconstructions at (a) 3 iterations and (b) 20 iterations. Table S1. This shows how bladder SUV affects lesion quantification for each lesion diameter. This is expressed as the % change in lesion SUV as bladder SUV increases. The SUV values are the mean values from all noise realisations at 30 full iterations with 4 mm post-filter. (DOCX 139 kb) [file 40658_2018_233_MOESM1_ESM.docx]

1. **Convergence property of the reconstruction algorithms**

For the convergence plot, we estimated the mean SUV_mean_ and SUV_max_ of the simulated lesions (L1 – L3) for bladder (SUV: 8.5 and 55.5) using the SUVs of the 20 noise realisations with 4 mm isotropic 3D Gaussian filter. The result is shown in Figure S1.

**Bladder SUV 8.5**

**Bladder SUV 55.5**

**Figure S1:** Convergence plots of all the reconstruction algorithms using lesion SUV_max_ and SUV_mean_. This is estimated for lesion diameter 10 mm at bladder SUV8.5 and 55.5, and it is obtained from the mean of all 20 noise realisations with 4 mm Gaussian post-filter. First, second and third columns represent the convergence plots for lesions L1, L2 and L3 respectively (each with true SUV: 8).

Each reconstruction algorithm, as well as each simulated lesion, seems to have slightly different convergence rates. Therefore, for SUV analysis in this study, the iteration number was increased to 30 so as to ensure a reasonable convergence of all algorithms and lesions. OSEM+PSF reconstruction has a higher SUV comparative with OSEM reconstruction both for SUV_mean_ and SUV_max_. This has been a commonly reported behaviour associated with the incorporation of PSF into the reconstruction [38, 39].

For L3 at bladder SUV55.5, OSEM and OSEM+PSF have higher SUVs at early iterations, but this reduces as the number of iteration increases. This might signify that for proximal lesions to hot regions, the spill-in effect reduces with iteration. However, OSEM+PSF+BC has similar behaviour at both bladder SUV8.5 and SUV55.5.

1. **Dependence of spill-in effect on lesion size, post-filtering and spatial resolution**

Figure S2 shows the relationship between the spill-in effect (due to increasing bladder activity) and lesion size. As expected, there is an underestimation in lesion SUV for small diameter lesion, but the SUV increases as the diameter increases. However, for L3, this behaviour is further influenced by increasing bladder SUV (spill-in effect). For a fixed diameter, the lesion SUV increases as bladder activity increases, thereby increasing the bias. This is not so evident in L1 and L2.

L1

L2

L3

L1

L2

L3

**Figure S2:** The variation of lesions uptake (SUV_max_ and SUV_mean_) with lesion size and bladder SUV. This is obtained from the mean SUV of all noise realisations at 30 full iterations with 4 mm Gaussian post-filter. The error bars are the standard error of the mean (SEM) while the dashed horizontal line denotes the true simulated lesion SUV.

The % relative change in lesion (*l*) SUV $({\Delta SUV}_{l})$ as bladder SUV increases from SUV8.5 to SUV55.5 was also estimated using Eq. 1:

${\Delta SUV}_{l}\left( \% \right)=\frac{{SUV}_{l\left( 55.5 \right)}-{SUV}_{l\left( 8.5 \right)}}{{SUV}_{l\left( 8.5 \right)}}\times100$ (1)

where ${SUV}_{l\left( 8.5 \right)}$ and ${SUV}_{l\left( 55.5 \right)}$represent the lesion SUV at bladder SUVs 8.5 and 55.5, respectively.

The % relative change in lesion SUV is further influenced by the application of post-filter as shown in Figure S3. Lesion L3 showed the highest % relative change in SUV (both SUV_mean_ and SUV_max_) as post-filter FWHM increases, and with 5 mm post-filter, this relative change was as much as 49% and 9% for SUV_max_ and SUV_mean_ respectively. L1 showed a slight relative change but L2 showed no defined relative change in SUV (Details in Table S1).

**Figure S3:** The dependence of spill-in effect on post-filter. This was done with a single noise realisation at 30 iterations.

**Table S1:** This shows how bladder SUV affects lesion quantification for each lesion diameter. This is expressed as the % change in lesion SUV as bladder SUV increases. The SUV values are the mean values from all noise realisations at 30 full iterations with 4 mm postfilter.

| **Lesion Diameter (mm)** | **% Relative Change in Lesion SUV** | | | | | |
| --- | --- | --- | --- | --- | --- | --- |
|  | **L1** | | **L2** | | **L3** | |
|  | **SUV_max_** | **SUV_mean_** | **SUV_max_** | **SUV_mean_** | **SUV_max_** | **SUV_mean_** |
| 6 | 1.67 | 0.31 | 1.89 | 0.51 | 10.94 | 3.90 |
| 8 | 3.08 | 0.45 | 1.41 | 0.85 | 15.09 | 4.90 |
| 10 | 3.37 | 0.39 | 1.99 | 0.70 | 30.67 | 5.77 |
| 12 | 4.96 | 0.33 | 2.07 | 0.99 | 53.53 | 8.50 |

To further investigate the spill-in effect as a function of the system resolution, instead of blurring the simulated PET images with the GE Signa PSF, the images were blurred with an isotropic Gaussian filter of FWHM ranging from 3 – 6 mm before forward projection.

Figure S4 shows the estimated spill-in activity around the dilated bladder shells as resolution increases (using Eq. 2). The spill-in activity is expressed as the % activity difference in the dilated shells between the simulated and OSEM reconstructed images.

$edge mask=dilation\left( bladder mask, n \right)- bladder mask$ (2)

where $dilation\left( bladder mask, n \right)$ means dilating the bladder mask by n voxels.

(**NOTE:** All equations are properly described in the main manuscript).

FWHM of 6 mm has the highest % activity difference, and this reduces significantly as resolution improves, with FWHM of 3 mm having the least % activity difference. Also, as expected, the immediate shell around the bladder (2 voxels) has the highest activity difference for all resolutions, but it reduces as we move further away. However, for FWHM of 5 and 6 mm, the activity difference remains almost constant and considerably high even at voxels 6 to 10 away from the bladder.

**Figure S4:** The spill-in activity from the bladder to the surroundings as a function of the system resolution (in FWHM), using Eq 2. The activity difference is estimated from a single noise realisation of OSEM reconstruction with bladder SUV 55.5 at 30 full iteration with 4 mm post-filter.

1. **Investigating the impact of TOF reconstruction in mitigating spill-in effect**

In order to explore the benefit of TOF reconstruction in correcting for spill-in effect, we reconstructed the NEMA IQ phantom using the GE PET Toolbox with and without TOF implementation (with 28 subsets and 20 full iterations). The reconstructed images have 256$\times$256$\times$89 voxels with size 2.34$\times$2.34$\times$2.78 mm^3^. We estimated the activity in the dilated shells surrounding the hot bottle using the morphological operation in Eq 2:

Figure S5 shows the normalised mean activity in each of the dilated shell surrounding the hot NEMA bottle, for TOF and non-TOF reconstructions. At 3 iterations, there is a slight improvement of TOF reconstruction over non-TOF reconstruction for 2 voxels around the bottle, however, at 20 iterations, the improvement is only seen at 1 voxel.

**(a)**

**(b)**

**Figure S5:** The normalised mean values within the dilated shells surrounding the NEMA bottle obtained at with (a) 3 iterations and (b) 20 iterations., with non-TOF and TOF reconstructions. The reconstructions were done with the GE PET toolbox.The voxel activity were normalised with the actual activity in the spheres.

It is worthy of note that at 2 voxels, the spill-in activity in non-TOF image is up to 17% at 3 iterations, but only 12% at 20 iterations. This also reiterate the fact that SUV overestimation as a result of spill-in effect reduces over iteration. Therefore, in order to reduce the spill-in effect, slightly increasing the number of iteration might be a good alternative. However, this will be at the expense of reduced CNR and increased noise.

For the NEMA bottle phantom, the spill-in activity from the hot bottle to the surrounding shells reduces as we move further away from the bottle, and this is lower for TOF reconstruction than non-TOF. Similar trend is also seen in the patient data (Figure S5), but major improvement with TOF reconstruction over non-TOF reconstruction in terms of reduced spill-in effect around the segmented spleen is visible only at higher iterations.

**(a)**

**(b)**

**Figure S6:** Normalised mean values within the dilated shells surrounding the spleen obtained with non-TOF and TOF reconstructions at (a) 3 iterations and (b) 20 iterations. The reconstructions were done with the GE PET toolbox. The voxel activity were normalised with the mean activity in the liver.
